# Tough Clinical Decisions: Experiences of Polish Physicians

**DOI:** 10.1007/s10730-022-09491-x

**Published:** 2022-08-08

**Authors:** Joanna Różyńska, Jakub Zawiła-Niedźwiecki, Bartosz Maćkiewicz, Marek Czarkowski

**Affiliations:** 1 Center for Bioethics and Biolaw, Faculty of Philosophy, University of Warsaw, Krakowskie Przedmieście 3, 00-047 Warsaw, Poland; 2 Collegium Medicum Cardinal Stefan Wyszyński University in Warsaw, ul. Kazimierza Wóycickiego 1/3, 01-938 Warsaw, Poland

**Keywords:** Ethical dilemmas, Clinical practice, Physicians, Poland

## Abstract

The paper reports results of the very first survey-based study on the prevalence, frequency and nature of ethical or other non-medical difficulties faced by Polish physicians in their everyday clinical practice. The study involved 521 physicians of various medical specialties, practicing mainly in inpatient healthcare. The study showed that the majority of Polish physicians encounter ethical and other non-medical difficulties in making clinical decisions. However, they confront such difficulties less frequently than their foreign peers. Moreover, Polish doctors indicate different circumstances as a source of the experienced problems. The difficulties most often reported relate to (i) patients (or their proxies) requests for medically non-indicated interventions; (ii) problems with communication with patients (or their proxies) due to the patients’ negative attitude, unwillingness to cooperate, or aggression; and (iii) various difficulties with obtaining informed consent. Polish physicians report difficulties associated with disagreements among care givers or scarcity of resources less frequently than doctors from other countries. The study’s findings provide support for the thesis that a significant portion of Polish physicians still follow a traditional, paternalistic, and hierarchical model of healthcare practice. Instead of promoting patient’s empowerment, engagement, and rights, they often consider these ideas as a threat to physicians’ professional authority and autonomy. The study leads to the conclusion that due to insufficient training in medical ethics, communication skills, and medical law, many Polish physicians lack the knowledge and competence necessary to adequately respond to challenges posed by modern healthcare practice.

## Introduction

Medicine, no matter how scientifically and technically advanced, has always been embedded in a moral context. It is due to its three salient features. Firstly, medicine is a professional practice aimed at promoting human life and health, i.e., goods highly valued by all people and societies because of their foundational role for the well-being of individuals and populations. Secondly, it is a social practice always immersed in a specific socio-economic, cultural, political and technical reality, and its complex normative fabric (Baker and McCullough 2008). This social reality evolves and re-shapes a moral framework for healthcare practice (Miller & Brody, 2001). This framework is usually described by a set of general moral principles, role-specific duties and clinical virtues, which together determine what constitutes an ethically sound relationship between patients and healthcare professionals. In liberal-democratic societies there is a widespread agreement that modern medical ethics is governed by four main moral principles: respect for patient autonomy, beneficence, nonmaleficence and justice/fairness (Jonsen et al., 2015; Beauchamp & Childress, 2019). Although, alternative sets of principles have been proposed in the literature as well (Veatch, 2007). For example, many European bioethicists argue for enriching the above four-principles list with respect for human dignity, integrity, vulnerability, and principles of precaution and solidarity (Rendtorff, 2002; Häyry, 2003; Kemp & Rendtorff, 2008). Whatever the proposed list of principles for medical ethics is, ranking, balancing, specifying, and implementing those principles into real-life clinical scenarios is not an easy task (Veatch, 1995; Richardson, 2000; Jonsen et al., 2015; Beauchamp & Childress, 2019). The modern clinical setting is a complex and dynamic environment marked with unprecedented advances in biomedical sciences, technologies and healthcare innovations, rising costs of healthcare systems complexities and resulting financial constraints, and the growth of both social expectations and socio-economic inequities in access to healthcare. All these factors make the medicine of today an extremely ethically challenging social and professional practice.

Thirdly, providing healthcare is an inherently humanistic and value-laden activity, because every clinical encounter happens between humans – patients and healthcare professionals – who are rational, but also emotional and moral beings. The parties’ values and interests are often aligned, as they are usually committed to pursue shared goals. However, since modern societies are morally fragmented and diverse, sometimes patients and clinicians’ viewpoints diverge or come into conflict. If that happens, uncertainties or conflicts regarding value-laden aspects of healthcare decision-making emerge. Such situations are a vital part of healthcare experienced by medical professionals worldwide.

There is an increasing amount of literature on ethical challenges faced by healthcare professionals in everyday practice. Most of the published papers focus on experiences of physicians and nurses practicing in countries with well-functioning liberal-democratic governance, strong civil societies, and – on average – good quality of healthcare, such as the United States (Gramelspacher at al. 1986; Walker et al., 1991; DuVal et al., 2004; Hurst et al., 2005), Canada (Gaudine et al., 2011a, b) or western and northern European countries (Bremberg & Nilstun, 2001; Hurst et al., 2007; Gjerberg et al., 2010; Jox et al., 2010; Oerlemans et al., 2015; Rasoal et al., 2016). The need for comparative data from countries with different sociopolitical histories and healthcare systems, especially those from central and eastern Europe, has been strongly emphasized in the literature (Hurst et al., 2007; cf. Sorta-Bilajac et al., 2008; Grosek et al., 2020; Grosek et al., 2021). This paper responds to this need by reporting results of the very first empirical study on Polish physicians’ experiences with – what we have called – *tough clinical decisions*, i.e., decisions about the patient’s treatment or care that Polish physicians consider difficult to make for moral reasons or other reasons not directly related to their medical knowledge or skills, e.g., communication or other interpersonal problems, limited resources, organizational issues, or legal requirements (Czarkowski et al., 2021; Kälvemark et al., 2004). Such decisions cause unease or uncertainty about what is right or good to do in a given clinical situation, and create a demand for an adequate clinical ethics support in clinical setting. Our purpose here is to identify and discuss non-medical circumstances of clinical decision-making that raise difficulties for Polish physicians. By making them explicit, we hope to provide an insight into the specific nature of tough clinical decisions faced by healthcare professionals from a culturally, politically, and economically transitional country – Poland.

## Methods

### Survey Questionnaire Development and Design

We present part of the findings from the very first comprehensive survey-based quantitative study on tough clinical decisions encountered by Polish physicians. The study covered numerous problems: (i) physicians’ experiences with tough clinical decisions (including their prevalence, frequency and context); (ii) ethical (and other non-medical) issues that contributed to these clinical decisions being tough; (iii) methods employed in dealing with those decisions; (iv) respondents’ ethical education and its perceived helpfulness; and (v) respondents’ knowledge and experience with clinical ethics consultation services. Issues (iii-v) are not reported in the present paper, as they have been already presented elsewhere (Czarkowski et al., 2021) or will be covered fully in separate reports.

The questionnaire developed for the study included 17 close-ended questions. Three of them included options labeled "other; please specify”. In questions regarding the perceived frequency or helpfulness of a given practice quasi-Likert 5 level scales were used. Some of the questions were interrelated and multilayered. While designing the questionnaire, we reviewed similar research from other countries for inspiration and comparability. The main inspiration came from a survey of U.S. internists’ experiences with ethical dilemmas (DuVal et al., 2004) and similar European studies (Hurst et al., 2007; Sorta-Bilajac et al., 2008, 2011).

As already mentioned, this report focuses on findings from an analysis of a set of questions regarding the prevalence, frequency and nature of ethical and other non-medical problems encountered by Polish physicians. The problems listed in the questionnaire included: uncertainty regarding patient’s decision-making capacity; problems with determining a legitimate surrogate decision-maker for an incompetent patient; patient’s (or her proxy’s) objection; patient’s (or proxy’s) request for medically non-indicated intervention; patient’s (or her proxy’s) request for an intervention the physician considers immoral (conscientious objection); patient’s (or her proxy’s) request for an intervention the physician considers unacceptable for other reasons (e.g., legal or administrative); uncertainty whether to disclose bad diagnosis or prognosis; uncertainty whether to maintain confidentiality (therapeutic privilege); disagreement among healthcare givers; necessity to ration scarce healthcare resources; and problems with communication with a patient (or her proxy). The list contained an open-ended option labelled “other, please specify”. Some of the answers provided by respondents for this open-ended question fitted within the original categories and were reassigned correspondingly. Additionally, on the basis of the obtained answers, three new categories of problems were defined: problems with futility and limitation of life-sustaining treatment; uncertainty whether to involve social services (social care, police) or court due to the difficult personal or family situation of the patient; problems with organizational limitations of professional autonomy of the physician.

The questionnaire was designed to be self-administered. During the development phase, a small pilot study with fifteen participants was conducted to assess the accessibility and understanding of the questionnaire, as well as the duration and feasibility of self-administration. Feedback from the pilot was incorporated into the questionnaire design. The overall length of questionnaire was reduced to fit within 10 minutes completion time, which was deemed the maximum acceptable by potential physician-respondents.

The study was conducted in the Polish language. Results reported herein have been translated into English. Translation was agreed on by consensus between the researchers. All authors have extensive education, teaching and research experience in English-speaking environments.

### Survey Distribution and Data Collection

The survey was advertised through multiple channels to all physicians with license to practice in Poland (approx. 150,000). These included the website of the Center for Bioethics and Biolaw of the University of Warsaw, where the study was affiliated, and the official newsletter and newspaper of the Polish Chamber of Physicians and Dentists. The Chamber distributes these papers to all registered physicians as one of its statutory duties. Medical conferences, training courses for healthcare professionals, and personal communication with individual physicians and hospitals’ administration were used for distribution as well. The questionnaire was available in two media: online and traditional pen and paper.

In the study period between June 2018 and February 2019, a total of 521 completed questionnaires were obtained with more than two thirds filled online (n = 289).

### Respondents

Of the total of 521 physicians who completed the survey, 43.8% (n = 228) already completed training in at least one medical specialty (referred to as specialists) and 50.1% were in a residency program in some specialty (further referred to as residents). We received only 32 surveys from M.D.s without specialty and not in residency training (referred to as “non-specializing physicians”). Most medical and surgical – both clinical and non-clinical – specialties were included in the sample (taking residents into account).

Due to a large number of official specialties in Poland (77) and a limited study sample, we decided to group specialties into major categories, as suggested by ordinances of the Ministry of Health of the Republic of Poland (2019, 2020): internal medicine, anesthesia and intensive care, pediatrics, psychiatry, family medicine, surgical specialties including gynecology, and other (mostly diagnostic, e.g., pathology, radiology, laboratory medicine). For the purpose of statistical analyses, we decided to create a “multispecialist” category that included physicians who had two or more specialties. These physicians were reported separately in order to avoid exclusion, as they were expected to be the most experienced respondents working in the most demanding clinical environments.

### Demographics

Due to the data collection technique, this sample is an opportunity sample and as such is not representative of the specialty structure or other demographics of Polish physicians.

In our sample, the average seniority was 11.24 years (SD = 10.55, MED = 7) with professional experience between less than 1 year to 54 years. Majority of participants had less than 20 years of seniority (79.6%). Only 20.4% (n = 106) had seniority of over 20 years. Interestingly, it was discovered that the pen and paper version was filled by significantly more experienced physicians than the online version (10.6 years (SD = 10.4, MED = 7) as compared to 13.1 years (SD = 10.7, MED = 8), Mann-Whitney U: U = 21,216, p = 0.0036).

In response to comments from the pilot study, which pointed out problems with anonymity in less common specialties and specialty combinations, data on age and gender was not gathered in the final questionnaire.

The majority of respondents worked in inpatient healthcare 86.6% (n = 451), with 82.7% (n = 431) working in hospitals. While 45.7% (n = 238) worked in outpatient settings e.g. clinics or private practice, only a small minority (13.4%; n = 70) worked exclusively in outpatient healthcare. These data do not sum up to 100%, as the majority of respondents hold multiple jobs across inpatient and outpatient settings.

### Data Analysis

The online survey was run using Limesurvey software. Data was analyzed in R programming environment. Statistical tests apart from descriptive statistics included Mann-Whitney U test and multiple logistic and linear regression. The tests used and the significance are indicated together with the corresponding results. “SD” is used for standard deviation, “M” for mean, “MED” for median, “OR” for odds ratio and “CI” for 95% confidence interval, where appropriate.

### Ethical Considerations

The survey did not involve the collection of personally identifiable information (personal data). Participation was anonymous and voluntary. The Research Ethics Committee at the Faculty of Sociology of the University of Warsaw approved the study.

## Results

As we have already reported (Czarkowski et al., 2021), the majority of study participants (76.6%; n = 399) encountered tough clinical decisions in the course of their practice. The odds of encountering such difficulties increased together with an increased level of professional knowledge, i.e., specialization status, even when controlled for possible confounding factors, such as size of community and type of clinical practice (inpatient v. outpatient), as presented in Table 1.

**Table 1:** *Multiple logistic regression model for factors influencing the odds of facing tough clinical decisions. For professional status we adopted “non-specializing physicians” as a reference level. For work setting we adopted “inpatient setting” as a reference level*

Predictor	statistic	OR [95% CI]	*p*
(Intercept)	0.189	1.073 [0.516–2.250]	0.850
Resident	2.743	2.882 [1.341–6.152]	0.006
Specialist	3.742	5.724 [2.301–14.458]	< 0.001
Experience (years of)	-0.997	0.986 [0.959–1.014]	0.319
Outpatient only	-2.919	0.431 [0.246–0.765]	0.004

A major increase in the odds of facing tough clinical decisions was especially visible among specialists, even when accounting for professional experience. To compare physicians with different areas of clinical practice to the whole sample, we fitted the logistic regression model with encountering tough clinical decisions as a binary target variable. The predictor variable (speciality category) was entered into the model using deviation coding. The same strategy was used for subsequent analyses of differences related to categories of specialization. The analysis shows that specialists (and residents) in anesthesiology and intensive care (OR = 3.095, *p* = 0.008), and respondents with specialty (or training) in internal medicine (OR = 1.939, *p* = 0.019) are more likely to encounter tough clinical decision. In fact, a vast majority of specialists in those areas encountered ethical issues (91.94% and 87.72%, respectively).

Among those respondents, who faced tough clinical decisions in the course of their practice, only slightly over 18% indicated that such situations occurred frequently (15.8%; n = 63) or very often (2.3%; n = 9). Almost 60% of them (n = 241) declared that they had faced such decisions very rarely (16%; n = 64) or rarely (44.5%; n = 177). However, the frequency of facing tough clinical decisions increased with the respondents’ professional status. The answers from quasi-Likert scale from “Never” to “Very often”, when transcoded into numbers from 1 to 5, produce mean values as presented in Table 2.

**Table 2:** *The frequency of encountering tough clinical decisions ranging from “Never” (1) to “Very often” (5)*

Professional status	M	SD	n
Non-specializing physician	1.219	1.476	32
Resident	1.885	1.368	261
Specialist	1.925	1.320	228

The multiple linear regression analysis showed that the increase in tough clinical decisions frequency is significant for both residents and specialists as compared to the non-specializing physicians. At the same time, there is also a very small but significant reduction in frequency of ethical (and other non-medical) problems encountered by respondents when considering years of experience. As with odds of encountering problems, the frequency is lower for physicians working exclusively in outpatient care (Table 3).

**Table 3:** *Multiple linear regression model for factors influencing the frequency of encountering tough clinical decisions. For professional status we adopted “non-specializing physicians” as a reference level. For work setting we adopted “inpatient setting” as a reference level*

Predictor	b [95% CI]	*p*
(Intercept)	1.056 [0.580–1.531]	< 0.001
Resident	0.717 [0.228–1.207]	0.004
Specialist	1.190 [0.642–1.738]	< 0.001
Experience (years of)	-0.026 [-0.041 - -0.011]	< 0.001
Outpatient only	-0.535 [-0.877 - -0.194]	0.002

When data was analyzed by specialty of respondents (multiple linear regression model with specialty category as a deviation-coded factor), specialists and residents in anesthesiology and intensive care (b = 0.731, p < 0.001), internal medicine (b = 0.386, p = 0.002) and psychiatry (b = 0.442, p = 0.044) were found to report facing difficult clinical decisions most frequently.

When asked about the areas of medicine in which they had faced tough clinical decisions, the respondents answered with the following distribution (Table 4).

**Table 4:** *Areas of clinical practice and the prevalence of tough clinical decisions*

Area of clinical practice	n (total = 521)	%*
Palliative and end-of-life care	232	44.5
Intensive care	198	38
Oncology	126	24.2
Neonatology	65	12.5
Gynecology and obstetrics; fertility treatment	54	10.4
Transplantations	34	6.5
Pediatrics	15	2.9
Psychiatry	14	2.7
Other	20	3.8

* The percentages do not sum up to 100, since the respondents could indicate more than one area of clinical practice

An analysis by medical specialty, professional status, seniority, and type of practice (in- v. out-patient setting) was generally consistent with the above-reported findings as well as with commonsensical assumptions that physicians trained in a certain specialty have higher odds of encountering ethical (and other non-medical) problems in their own field; and that physicians who practice in outpatient setting only do not or very rarely face problems associated with healthcare interventions offered in inpatient care exclusively (e.g., intensive care, neonatal intensive care, transplantations).

Respondents were also asked to indicate sources of ethical (and other non-medical) difficulties they had encountered in their practice. The answers are presented in Table 5.

**Table 5:** *Circumstances leading to tough clinical decisions*

Category	n (total = 521)	%*
Request for medically non-indicated intervention	262	50.3
Communication with a difficult patient (her proxy)	234	44.9
Uncertainty about the patient’s capacity	213	40.9
Patient’s (proxy) objection	174	33.4
Identification of a legitimate surrogate decision-maker	147	28.2
Disagreement among healthcare givers	145	27.8
Necessity to ration resources	130	25
Uncertainty whether to maintain confidentiality	127	24.4
Uncertainty whether to disclose diagnosis or prognosis	94	18
Request for otherwise unacceptable intervention	81	15.5
Conscientious objection	73	14
Futility and limitation of life-sustaining treatment	20	3.8
Uncertainty whether to involve social services or court	12	2.3
Organizational limitations of professional autonomy	12	2.3

* The percentages do not sum up to 100, since the respondents could indicate more than one category of circumstances leading to tough clinical decisions

Analysis by professional status, seniority and place of practice did not yield results diverting from general trends. When analyzed by specialty, some significant results were observed. Looking at the categories one by one, both specialists (residents) in anesthesiology and intensive care (OR = 1.559, *p* = 0.075) and specialists (residents) in internal medicine (OR = 1.502, *p* = 0.040) were significantly more likely to have problems with requests for medically non-indicated interventions. Also, patient’s uncertain capacity was more likely an issue for anesthesiologists and intensivists (OR = 3.586, *p* < 0.001) and internal medicine physicians (OR = 2.259, *p* < 0.001), while it was not significantly more likely to be problematic for psychiatrists (OR = 1.689, *p* = 0.124). None of the specialties were outstanding in encountering difficulties with communication. Maintaining confidentiality and or rationing scarce medical resources were the most problematic for psychiatrists (OR = 2.607, *p* = 0.004). Specialists (and residents) in pediatrics were significantly less likely to have problems with confidentiality (OR = 0.299, *p* < 0.001) and patient’s capacity (OR = 0.149, *p* < 0.001) than other specialists, while being more likely to have problems with disagreements among healthcare givers (OR = 2.379, *p* < 0.001) and patient’s objection (OR = 1.498, *p* = 0.060). At the same time, identification of a legitimate surrogate decision-maker wasn’t likely a frequent issue for them (OR = 0.724; *p* = 0.211). The latter was more likely to be problematic for internal medicine specialists (residents) than for all other specialists (OR = 1.908, *p* = 0.002). Internal medicine physicians also stood out in facing uncertainty whether to disclose diagnosis or prognosis, i.e., therapeutic privilege (OR = 1.788, *p* = 0.023).

## Discussion

This is the very first survey assessing the prevalence and nature of ethical and other non-medical dilemmas faced by Polish physicians in their everyday clinical practice. The study shows that most Polish physicians encounter tough clinical decisions, i.e., decisions about the patient’s treatment or care which they found difficult to make for ethical or other non-medical reasons (e.g., due to interpersonal, cultural, organization, or legal problems). Similar to physicians in other countries, Polish doctors indicated higher likelihood of encountering such difficulties if they had specialty training, in particular in anesthesiology and intensive care or internal medicine/oncology, or worked in inpatient care (DuVal et al., 2004; Hurst et al., 2007; Saarni et al., 2008).

However, this study also reveals that Polish physicians are less likely to face or admit facing tough clinical decisions than their peers from the United States and western or northern European countries. Slightly above three quarters of our respondents (76.6%) reported confronting tough clinical decisions, whereas in similar studies with US physicians practicing general internal medicine, hematology/oncology, or critical care/pulmonary medicine (DuVal et al., 2004), general practitioners from Great Britain, Italy, Norway, and Switzerland (Hurst et al., 2007) and physicians from Croatia and Slovenia (Grosek at al. 2020; Grosek et al., 2021), almost all surveyed doctors (90% or above) reported having such an experience. Moreover, only slightly over 18% of Polish physicians, out of those who faced a tough clinical decision at least once, indicated that such situations occurred frequently or very often, whereas in the quoted European survey over 61% of physicians from Great Britain, 48% of Swiss doctors, 44% of doctors from Norway and 36% from Italy declared they faced ethical difficulties often (Hurst et al., 2007).

Why do Polish physicians report less ethical problems? There seems to be at least six possible explanations of this situation, however, they all require further analysis and research.

Firstly, the low experience with tough clinical decisions may stem from the larger diversity of medical specialties represented by the surveyed Polish physicians in comparison with the samples surveyed in the above-cited European and US studies. The diversity may translate into lower total or average results.

Secondly, it may result from a greater attachment of Polish physicians to a paternalistic model of the doctor-patient relationship. When the physician knows best and when the patient’s values and preferences are ignored or not explored, there is little chance for conflicts over the course of future treatment or care. This explanation finds support in the OECD/EU reports on European patients’ experiences with their primary care doctors in the ambulatory healthcare system (2016, 2018, 2020). The reports show that Polish physicians scored the lowest in Europe when it comes to respect for patients’ autonomy and communication. As reported by OECD/EU in 2016, less than one in two Polish patients (47.9%) admitted having been involved in decision-making regarding their care and treatment, while across the countries under comparison the average rate was 78.3%. Additionally, only one in three Polish patients (33.6%) confirmed having been given the opportunity to ask questions or raise concerns, and less than 2/3 (59.6%) reported that their primary care physicians spent enough time with them in consultation. In contrast, average scores for analogous patients’ experiences with primary care across all European countries under investigation were 83.2% and 82.2%, respectively. It is worth noting, however, that the 2020 OECD/EU report noted progress in the percentage of Polish doctors involving patients in care/treatment decisions (61.5%).

Thirdly, the lower incidence of reported ethical and non-medical problems can result from poor ethical training of Polish physicians resulting in their insufficient knowledge and skills in identifying and, what follows, acknowledging ethical as well as social, cultural, organizational and legal dimensions of healthcare decision-making. Our respondents could have encountered tough clinical decisions much more often that they in fact realized or wanted to realize. This explanation is consistent with results of another part of our study, which will be fully reported elsewhere, on the practical usefulness of ethical education during formal medical education, on both graduate and postgraduate levels. We established that most of the surveyed Polish physicians (83%; n = 424) had taken a course in medical ethics or bioethics in medical school, and almost half of them (41.2%; n = 211) had participated in some type of trainings in ethics during their post-graduate medical education. However, over 60% of them found the received ethical education deprived of any usefulness in solving ethical dilemmas in their daily practice. Only one in five respondents evaluated their ethical education to be helpful in this respect. The results were better for ethical education offered at the post-graduate level, i.e., during specialty trainings. Almost 45% of the surveyed Polish physicians found post-graduate courses in medical ethics or bioethics to be useful in their current clinical practice. However, still almost 40% of the respondents denied the ethical education any practical value and usefulness. These findings highlight the need for improvement of ethical education offered to Polish medical students and medical professionals.

Fourthly, “insensitivity” to difficult ethical (or other non-medical) aspects of healthcare practice can be acquired in the process of informal and tacit modes of secondary socialization (so called “hidden curriculum”) by which professional cultures are communicated to new members, i.e., medical students and young physicians (Hafferty & Franks, 1994; Vaidyanathan 2015). When senior physicians, i.e., the main agents of the professional socialization, act paternalistically towards their patients, stick to the rigid hierarchical authority structure of the medical teamwork and healthcare decision-making, and when they present themselves as being desensitized and morally detached, there is a high risk that their younger colleagues will mimic these emotional and behavioral patterns in their future professional life (Lind, 2000; Hren et al., 2011; McDonald et al., 2021). The revealed reduction in frequency of ethical (and other non-medical) problems encountered by Polish physicians, when considering their years of experience, provides support for this explanation.

Fifthly, ignoring ethical, social, legal and other non-medical complexities associated with clinical practice may be one of mechanisms of reducing moral distress (Ulrich & Grady, 2018), or even an effect of professional burnout (Mangory et al., 2021; Passalacqua et al. 2012, Drummond, 2015). Although data are scarce, both phenomena are vivid among Polish physicians and urgent need to address them by adequate preventive and therapeutic measures is recognized (Zgliczyńska et al., 2019; Owoc et al., 2021).

Last but not least, lower reporting rate of tough clinical decisions by Polish physicians may be related to the fact that Polish law does not allow for performing certain potentially ethically controversial medical interventions, such as euthanasia, physician-assisted suicide, surrogacy, or abortion on demand, which are legally recognized in many European countries and US state legislations. Moreover, many legal instruments used in other countries, such as advance directives or medical proxy, remain unregulated in Polish law, thereby discouraging both patients and medical professionals from using them.

Polish physicians, who reported experience with tough clinical decisions, most frequently indicated end-of-life/palliative care (44.5%), anesthesiology and intensive care (38.0%), and oncology (24.2%) as the most ethically challenging areas of clinical practice. This should come as no surprise as these fields of healthcare involve terminally ill patients or individuals on the verge of life and death, often with limited or severely compromised capacity to make decisions for themselves. Providing medical help to such patients inevitably raises ethical, social, and legal questions. Numerous empirical studies support this observation (Oberle & Hughes, 2001; Torke et al., 2009; Gjerberg et al., 2010; Jox et al., 2010; Schildmann et al., 2011, 2013; Oerlemans et al., 2015; Hernández-Marrero et al., 2016). For example, in the above-cited survey of European physicians’ experiences with ethical dilemmas led by Hurst et al., (2007), two out of three of the most often reported ethical difficulties were associated with treating patients with impaired or uncertain capacity (94.8%), and limiting life-sustaining treatment at the end of life (79.3%). Also in a Croatian study, performed with the original questionnaire developed by Hurst et al., two most frequently reported ethical dilemmas involved uncertain or impaired decision-making capacity and limitation of treatment at the end of life. Those problems were indicated by 66% and 60% of the surveyed Croatian physicians respectively (Sorta-Bilajac et al., 2008). Similar results were obtained in a recent survey of Italian physicians. Two most commonly reported problems regarded treating patients with diminished decision-making capacity (70.4%) and providing treatment disproportionate due to the expected outcomes or patient’s condition (56.8%) (Leuter et al., 2018). Also, in the previously mentioned U.S. study involving internists, 65% of respondents admitted facing ethical dilemmas associated with incurable or dying patients, 39% reported problems with patient’s proxies, and 24% with requests for futile treatment (Hurst et al., 2005). In general, 51–78% of the surveyed U.S. doctors encountered difficulties with end-of-life decisions, and 35–61% reported issued with the patient’s decisional autonomy (exact percentages differed among subspecialties) (DuVal et al., 2004).

While in the quoted European and US surveys, the three most frequently reported ethical problems were related to decisions regarding end-of-life care, treatment of patients with diminished capacity, and disagreements among parties (DuVal et al., 2004; Hurst et al., 2005, 2007; Leuter et al., 2018), results of our study are interestingly different. The highest percentage of Polish physicians reported difficulties resulting from requests for medically non-indicated interventions (50.3%). Additionally, almost one third of the respondents reported problems with other patients’ (proxies’) requests which they found unacceptable for non-clinical (15.5%) or moral (14%) reasons. Since the physicians were not asked to provide any detailed information about the contested requests for medically non-indicated procedure, it may be assumed that they encompassed all kinds of patients’ (proxies’) pleas: from demands for futile treatment (in fact, almost 4% of respondents mentioned medical futility as a source of ethical difficulty in an open-ended question), through requests for redundant diagnostic or therapeutic procedures, to pleas for issuing undue sick leave certificates.

The second most common source of difficulties encountered by Polish physicians were communication issues caused by the patient’s (or proxy’s) behavior, negative attitude, unwillingness to cooperate, or aggression. In contrast, only 10% of the surveyed U.S. doctors indicated that they had experienced difficulties communicating with patients or their relatives, with members of the therapeutic team, or other providers of healthcare-related services, e.g., representatives of the insurer (Hurst et al., 2005).

These results suggest that the majority of Polish physicians perceive patients as “difficult”, i.e., demanding, angry, and uncooperative. Moreover, they suggest that the percentage of patients considered by Polish physicians as “problem-causers” is much higher than in studies involving clinicians from other countries, where approximately 15–30% of patients were perceived by doctors as difficult (Fischer et al., 2019; Hahn et al., 1996; Jackson & Kroenke, 1999; Hahn, 2001). Why is that so? We believe the results reveal that many Polish physicians do not adequately understand their professional obligations towards patients. They might also lack ethical and social competences and skills necessary to practice truly patient-centered care, despite the fact that “promoting more people-centered care has become a growing priority across EU countries in recent years to improve the quality of care and the responsiveness to patients’ expectations” (OECD/EU 2020, 182).

As shown above, Polish doctors are still heavily embedded in a paternalistic tradition of practicing medicine. They do not truly acknowledge that the modern patient is not a passive recipient of doctors’ advice and directives, but an active participant of the therapeutic process. Todays’ patients are aware of their rights and know how to claim them. They have a right to be provided with easy to understand and comprehensive information on available therapeutic options, right to ask questions and raise concerns, express their opinions and preferences, and make final decisions regarding future course of their treatment or care. Moreover, due to the unprecedentedly wide access to medical knowledge (both in scientific literature and in popular mass media, including internet) as well as to information on the comparative quality and availability of healthcare services offered by other providers (public or private, local or foreign), contemporary patients are not medically ignorant, at least in their own view. They often come to a doctor’s office with certain expectations regarding their diagnosis and future treatment, which might be medically correct, but may also be entirely unsubstantiated or unrealistic (e.g., due to the growing problems of Polish public healthcare system – its under-financing, long waiting lists, deficient infrastructure and equipment, etc.), *ergo*, impossible to fulfill by the healthcare professional. The latter is often the case with patients suffering from life-threating, chronic or incurable disease (and their families), who are desperately in need of hope and, because of that, particularly vulnerable to fake, misleading and ill-informed medical advice. Modern physicians should be responsive to and prepared for dealing with patients’ expectations – both realistic and unfounded or exaggerated – as well as their emotions, including anxiety, fear, disappointment, frustration, dissatisfaction, or anger.

The results of our study show that many Polish physicians lack ethical and interpersonal competences essential for engaging in respectful dialogue and shared decision-making process with patients and their families. Consequently, they treat patients paternalistically or perceive them as “difficult”. A recent study conducted by Gedeon Richter Poland, as a part of its education program “Recipe for Success in Medicine”, revealed that over 40% of the surveyed Polish doctors were afraid of patients – of their excessive expectations, the possibility of filing a lawsuit, and their potential aggressive behaviors (Lang, 2019). Almost half of the respondents stated that they needed additional education in medical law (47%) as well as in psychology and interpersonal communication (43%) These postulates are not new. The need to provide medical professionals with adequate training in communication skills has been highlighted for many years. However, only in 2015, the Polish Society for Medical Communication was created. In 2021, the Society issued the very first recommendations about communication skills training for medicine and dentistry students (Borowczyk et al., 2021).

The third group of ethical and other non-medical problems encountered by Polish physicians participating in our survey related to the process of obtaining valid informed consent. Circa 2/5 of the respondents (40.9%; n = 213) experienced uncertainty about the patient’s decision-making capacity. Almost 1/3 (28.2%; n = 147) had problems identifying a legitimate surrogate decision-maker for a patient who – due to her age, health status, or other reasons – was unable to give consent. A similar percentage of the study participants (33.4%; n = 174) reported difficulties with the patient’s (or her proxy’s) objection to the proposed healthcare intervention. In total, consent-related circumstances accounted for 31% of the reported difficulties.

With the exception of patient’s objection, at first glance these results may come as a surprise when interpreted against the paternalistic character of the Polish healthcare practice. Paradoxically, however, they are consistent with the finding that many Polish doctors perceive patients as “difficult” – demanding and hostile. Although more research is needed, these data seem to suggest that many Polish physicians still treat the requirement to obtain informed consent merely as a formality, a “paper” that will protect them from exposure to legal liability, rather than an ethical imperative aimed at protecting the patient’s autonomy, inviolability, and right to self-determination. The results of the above-cited study by Gedeon Richter support this presumption. Almost half of the doctors who participated in this survey (43%) admitted that they were afraid of the possibility of a patient filing a lawsuit and bringing a lawsuit, although few actually faced such situation in their medical practice (Lang, 2019).

The discussed findings also show that almost 1/3 of the surveyed Polish physicians (28,2%; n = 147) had problems with identifying a surrogate decision-maker authorized to make decisions regarding the treatment of an incompetent patient. This indicates the insufficient level of the respondents’ knowledge of the Polish medical law, which provides clear rules for substitute decision-making. However, it also highlights problems raised by the absence of specific legal regulations regarding the admissibility and validity of advance directives, including the appointment of healthcare proxy. The lack of statutory regulations in this respect creates numerous ethical and legal uncertainties for both physicians and patients.

Finally, it is worth noting that almost 1/3 of the surveyed Polish physicians reported tough clinical decisions due to disagreement among healthcare providers (27.8%; n = 145) and/or the necessity to ration scarce healthcare resources (24.9%; n = 130). These results are interesting, because they are significantly lower than in analogous European and northern American studies. Disagreements among care givers were a source of ethical difficulties for 81.2% of doctors from Norway, Switzerland, Great Britain and Italy (Hurst et al., 2007), 54% of the U.S. physicians (Hurst et al., 2005), 52% of Italian physicians (Leuter et al. 2008) and 47% of surveyed doctors from Croatia (Sorta-Bilajac et al., 2008; however, this data covers both disagreements between care givers and between family members). Most probably, the difference between Polish physicians and healthcare professionals from other countries results from a more hierarchical and authoritarian style of healthcare decision-making in Polish clinical practice. Despite deep political, socio-economic, organizational and regulatory changes in the Polish healthcare system that took place during the last three decades, the subordination and hierarchical dependency between the chief of unit and healthcare professionals working in the unit are still prevalent in Polish hospitals (Łuków & Wrześniewska-Wal, 2007; Krajewska, 2021). Chiefs of units retain substantial power and authority in the decision-making processes, including clinical ones. Like captains on ships, they usually have the final word on the course of patients’ treatment, although formally they are no longer authorized to give professional orders to their unit’s staff, only recommendations and guidelines (Łuków & Wrześniewska-Wal, 2007; Krajewska, 2021). The above-mentioned lack of appropriate respect for patients’ autonomy and the absence of laws regarding advance directives, including the appointment of healthcare proxy, contributes to the petrification of this situation.

Interestingly, despite the constantly exacerbating under-financing of the Polish public healthcare system, Polish physicians experience less problems with the rationing of scarce healthcare resources in comparison to their peers from western European countries. Scarcity of resources was reported by 40–60% of European doctors surveyed by Hurst et al., (2007), almost 54% of Italian physicians (Leuter et al., 2018), and more than 40% of Croatian doctors (Sorta-Bilajac et al., 2008; cf. Grosek et al., 2021). In a Slovenian study, ethical dilemmas associated with “long waiting periods for diagnostic and therapeutic treatment” and/or “allocation of limited resources” were indicated by 69.7% and 37.3% of the surveyed physicians, respectively (Grosek et al., 2020). However, only 20% of U.S. doctors indicated allocation as a source of ethical problems (Hurst et al., 2005). Due to the huge diversity of healthcare systems worldwide, these comparative results are not easy to interpret. Nevertheless, it seems safe to comment that the majority of Polish physicians get used to and accept the fact that their professional autonomy and best clinical judgment can’t always be pursued due to the system deficit and deficiencies preventing quality care.

### Limitations of the Study

The reported study suffers from several limitations (Czarkowski et al., 2021). Due to the methods of sample selection and questionnaire distribution, we were unable to recruit a representative sample of Polish physicians in regard to their age, professional status and experience, specialty and location of practice. There is an overrepresentation of younger respondents in the studied population, and underrepresentation of those working in rural environments, as well as those providing outpatient care only. The participants constitute an opportunity sample also in respect to represented medical specialties. However, the represented wide spectrum of medical specialties may be viewed as a strength of the study, as it provides insight into a larger variety of ethical and other non-medical difficulties encountered by Polish healthcare professionals.

More research is needed to identify actionable areas in physician education, training and working environment. This topic would benefit from both more focused studies on key practice areas and specialties, and qualitative research focused on mechanisms of the problems identified in this study. Studies designed to directly feed into planned reforms of healthcare and healthcare systems as well as professional education and training would be especially valuable.

## Conclusions

This paper reports findings of the prevalence, frequency and nature of ethical or other non-medical difficulties faced by Polish physicians. The study leads to the following conclusions supplemented with tentative recommendations that could be of use within Polish or similar healthcare systems.

1. The majority of Polish physicians encounter tough clinical decisions, i.e., problems with making clinical decisions due to ethical or other non-medical reasons (e.g., interpersonal, organizational, or legal) in their daily clinical practice. The odds of facing such decisions are significantly higher for specialists and doctors providing inpatient care.
  Recommendation: Readily available ethics support mechanisms need to be deployed through the healthcare system, but especially in highly specialist and inpatient services.

2) Similarly to physicians from western and northern Europe and the Unites States, Polish doctors face most difficulties while treating patients on the verge of life and death, and patients with limited or uncertain decision-making capacity. However, in contrast to their foreign peers, Polish doctors confront tough clinical decisions less frequently.
  Recommendation: Awareness of ethics issues needs to be improved. Focusing on real cases from daily practice in continued education might yield results.

3) Moreover, they indicate different circumstances as a source of problems with healthcare decision-making. The difficulties most often reported by Polish physicians relate to (i) patients’ (or their proxies’) requests for medically non-indicated interventions; (ii) communication problems due to patients’ (or their proxies’) negative attitude, unwillingness to cooperate, or aggression; and (iii) obtaining informed consent (assessing patient’s decision-making-competence; identifying an authorized surrogate; dealing with patient’s objection). Polish physicians report difficulties associated with disagreements among caregivers or scarcity of resources less frequently than doctors from comparative countries.
  Recommendation: Competences like communication skills – both within and without the healthcare team - should be included in continued medical education.

4) As discussed, these findings are in line with other data showing that many Polish physicians still follow a traditional, paternalistic, and hierarchical model of healthcare practice. Instead of promoting patient’s empowerment, engagement, and rights, they often consider these ideas as a threat to physicians’ professional authority and autonomy. For many of them, informed consent is merely a formal requirement aimed at protecting healthcare professionals against legal liability rather than an ethical foundation of patient-physician relationship.
  Recommendation: Serious reconsideration is required of the way informed consent is thought, presented and regulated in medical practice in Poland in order to change the “legal formality” view.

5) Additionally, due to insufficient training in medical ethics, communication skills, and medical law, many Polish physicians lack the knowledge and competence necessary to adequately respond to challenges posed by modern healthcare practice.
  Recommendation: More serious status needs to be attached to non-clinical competences of physicians that are indispensable for modern clinical practice, be it ethics, law or IT skills.﻿
